# Fire in the Belly? Sulfur-Reducing Gut Microbes Fuel Arsenic Thiolation

**DOI:** 10.1289/ehp.122-A222

**Published:** 2014-08-01

**Authors:** Carol Potera

**Affiliations:** Carol Potera, based in Montana, has written for *EHP* since 1996. She also writes for *Microbe*, *Genetic Engineering News*, and the *American Journal of Nursing*.

Inorganic arsenic, a ubiquitous environmental toxicant, is well known for its harmful effects in humans, including cancer, diabetes, and cardiovascular disease.^1^ Organic forms of arsenic, such as monomethylarsonic acid (MMA^V^), are generally considered less toxic than inorganic arsenicals. Researchers report in this issue of *EHP* that certain bacteria in the human colon can promote the conversion of MMA^V^ into the more toxic metabolite monomethyl monothioarsonic acid (MMMTA^V^).^2^

MMMTA^V^ is what’s known as a thiolated arsenical; “thiolated” means it contains a sulfur group. Thiolated arsenicals can be up to 100 times more cytotoxic than their non-thiolated counterparts.^3^ MMMTA^V^ and another thiolated metabolite, dimethyl monothioarsinic acid, have been detected in the urine of people who drank water contaminated with inorganic arsenic.^4^^,^^5^

**Figure:**
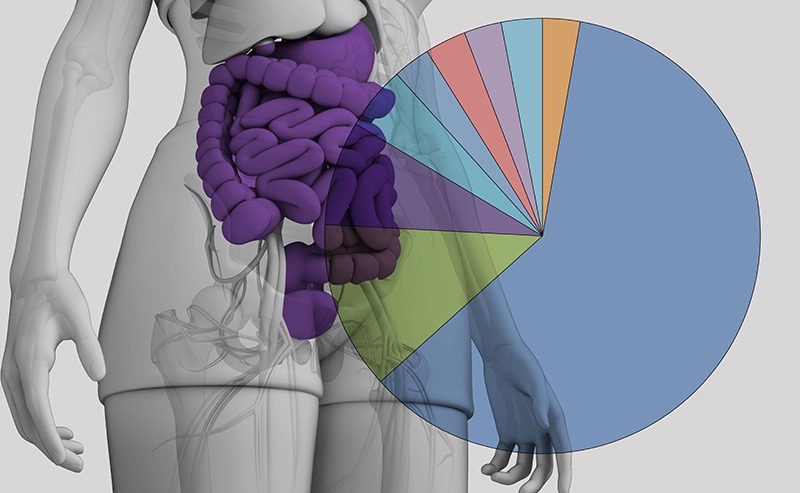
Gut microbe cultures with a large proportion of sulfur-reducing bacteria produced significantly more of a toxic arsenic metabolite than nonenriched cultures. This could help explain why some individuals are especially sensitive to arsenic’s harmful effects. © Shutterstock; DC.Rubin et al. (2014)^2^

The authors of the new study hypothesized that sulfur-reducing bacteria might be important for converting MMA^V^ to a thiolated form. They tested their hypothesis using a Simulator of the Human Intestinal Microbial Ecosystem (SHIME), a device that mimics the digestive processes of the stomach, small intestine, and ascending, transverse, and descending portions of the colon. SHIME “is an *in vitro* tool that helps to mechanistically explain *in vivo* observations. You can tweak certain parameters while keeping others constant,” says team leader Tom Van de Wiele.

A series of experiments were performed using fecal samples reflecting a full complement of gut microbiota, samples in which sulfur-reducing bacteria were either enriched or suppressed, and pure cultures of *Desulfovibrio desulfuricans (piger)*. The fecal samples were collected from 7 individuals, none of whom had taken antibiotics within the past 6 months.

The authors found that most arsenic biotransformation took place in the ascending and transverse colon. Hydrogen sulfide produced by gut bacteria drove this biotransformation; the addition of molybdate blocked hydrogen sulfide production and the conversion of MMA^V^ to MMMTA^V^. Fecal microbiota from the 7 individuals produced varying amounts of hydrogen sulfide, which corresponded with variations in MMMTA^V^ formation. Based on this evidence, the authors conclude that arsenic thiolation in the gut “can be considered a chemical process that requires a biological trigger, that is, sulfide production by metabolically active [sulfur-reducing bacteria].”^2^

The health consequences of the thiolated methylarsenicals produced in the gut remain unknown. “It’s an ‘orange flag,’ and one example of how certain microbial groups may contribute to increased toxicant risk,” says Van de Wiele. Numerous studies from the Human Microbiome Project have reported that the microbiome plays an integral role in human health.^6^ Similarly, toxicokinetics and pharmacokinetics also play important roles. “We cannot neglect these microbial processes,” says Van de Wiele.

Evaluating the potential risk of these compounds will not be a straightforward process; with an estimated 100 trillion microbes inhabiting the human gastrointestinal tract,^7^ the possible interactions with arsenic are endless. A first attempt could measure the conversion of MMA^V^ to MMMTA^V^ in human fecal samples. The results from the lowest and highest arsenic-converters may provide clues about how microbial processes control toxicant conversion, proposes Van de Wiele.

“The active involvement of sulfur-reducing bacteria in arsenic thiolation offers a novel intervention strategy to modulate arsenic metabolism by altering these bacteria,” says Kun Lu, an assistant professor at the University of Georgia, Athens, who was not involved with the study. Lu says the study also shows the clear impact of these bacteria on individual variability in thiolation, providing further insight into the role gut bacteria may play in individuals’ differing susceptibility to arsenic. Lu and colleagues have reported that infecting mice with *Helicobacter trogontum*, a potential cause of irritable bowel disease, changed not only the profile of the animals’ gut microbiomes but also the methylation and thiolation of arsenic metabolites excreted in urine.^8^
